# A study of the effects of four reading styles on college students’ mental health and quality of life based on positive psychology-A first-of-its-kind study

**DOI:** 10.1371/journal.pone.0308475

**Published:** 2024-08-28

**Authors:** Yamei Liu

**Affiliations:** Shanghai University Of Political Science and Law, Shanghai, China; Ahvaz Jundishapur University: Ahvaz Jondishapour University of Medical Sciences, ISLAMIC REPUBLIC OF IRAN

## Abstract

**Background:**

The increase in mental health problems among college students has become a global challenge, with anxiety and depression in particular becoming increasingly prevalent. Positive psychology has gained attention as an important psychological intervention that emphasizes improving mental health by promoting positive emotions and mindfulness. However, with the diversity of reading styles, however, there is a lack of systematic research on these effects. Therefore, this study aims to explore the specific effects of different reading styles on college students’ mental health and quality of life based on positive psychology, with the aim of providing more effective interventions and recommendations for improving college students’ mental health.

**Methods:**

This study used a two-round questionnaire to select students with mental health problems and divided them into four experimental groups with a control group. The study was conducted by distributing questionnaires and experimental interventions, and a total of 2860 valid questionnaires were collected. The study used the Self-Assessment Scale for Anxiety (SAS) and the Self-Depression Scale (SDS) to assess the participants’ anxiety and depression levels. In addition, the study used the Physical Composite Score (PCS) and the Mental Composite Score (MCS) to assess the participants’ quality of life. SPSS 26.0 was used for data statistics and repeated measures ANOVA was used.

**Results:**

Paper text reading and audio reading methods were effective in reducing anxiety levels and improving sleep quality. However, the electronic text reading approach was less effective compared to paper text reading and audio reading, and the video reading approach was not effective in improving depression. In addition, the positive psychology literature reading intervention showed significant improvements in college students’ quality of life scores.

**Conclusion:**

The results of this study suggest that paper text reading and audio reading modalities have a positive impact on the mental health and quality of life of college students, while e-text reading and video reading modalities are less effective. These findings provide suggestions for college students to choose appropriate reading styles and further demonstrate the effectiveness of positive psychology reading on mental health. These results have important academic and practical implications for promoting mental health and improving quality of life among college students.

## Introduction

Concern, and the problem of negative emotions among college students is becoming increasingly serious, with anxiety and depression becoming prevalent mental health challenges [1]. The college student population is particularly vulnerable to anxiety and depression due to factors such as academic stress, interpersonal relationships, and uncertain employment prospects [2–4]. A study by Stallman [5] noted that poor mental health in higher education globally has become a serious problem for public health. Li [6] found that mental health problems can lead to serious consequences such as extreme behaviors such as suicide. These studies emphasize the urgency and importance of mental health problems among college students. Mental health problems also affect the quality of daily life and sleep quality of university students, which negatively affects academic efficiency and life status [7]. A study by Ataei [8] showed that mental health problems such as anxiety and depression reduce life satisfaction and make it more difficult to enjoy life. This may manifest itself in the form of diminished interest in daily activities, reduced socialization, and even a loss of confidence and motivation in life. Mental health problems are also closely related to the quality of sleep and academic efficiency of college students. Studies by Alsubaie [9] have shown that mental health problems such as anxiety and depression can lead to decreased quality of sleep, which affects college students’ learning and memory. This reduced sleep quality may lead to daytime fatigue and poor concentration, which in turn affects academic efficiency and performance. In addition, negative mental states can seriously affect the quality of life, mainly in daily physical discomfort, as well as negative mental states in life [10]. Lovell’s [11] study showed that persistent negative emotions can lead to tension and stress responses in the body, increasing the risk of various physical illnesses, and that a long-term state of anxiety may lead to muscle tension, headache, stomach upset, and other physical discomfort, while chronic depressive states may be associated with physiological problems such as decreased immune system function and cardiovascular disease. In addition, negative psychological states can also have an impact on an individual’s habits and behaviors. Jenkins [12] showed that psychological problems such as depression and anxiety tend to lead to a decrease in an individual’s interest and motivation in daily activities, which in turn affects his or her quality of life. In addition, a study by Tavakoly [13] found that depressed individuals often lack active participation in daily life and may suffer from sleep disorders, eating disorders, and other problems, which further exacerbate their feelings of physical discomfort and negative psychological states. Therefore, timely and effective intervention and management of negative psychological states are essential to promote individuals’ physical and mental health and enhance their quality of life.

Positive psychology is a branch of psychology that emphasizes individual strengths, abilities, and resources and aims to improve the quality of life by enhancing positive emotions, individual growth, and psychological resilience [14] Scholars such as Martin Seligman proposed this theory in the early 1990s, arguing that psychology should focus not only on problems and deficits, but also on the positive aspects of individuals. They pointed out that through the cultivation of positive emotions, the development of individual strengths, and the search for meaning and purpose, individuals can better cope with the challenges and difficulties of life. Positive psychology emphasizes individual strengths and resources rather than problems or deficits, and enhances mental health and quality of life by promoting positive emotions and individual growth. In addition, positive psychology emphasizes an individual’s psychological resilience, which is how quickly an individual adapts and recovers in the face of challenges and adversity [15]. This resilience is not only the ability to cope with stress, but also the ability to learn, grow, and rebuild hope from setbacks and failures. By developing this psychological resilience, individuals can better cope with change and uncertainty in their lives, thereby maintaining mental health and a stable emotional state. In addition, positive psychology deals with how individuals find and realize meaning and purpose in life. It is believed that individuals having clear goals and perceived meaning in life can help them to be more motivated to pursue their desires and ambitions. This pursuit not only enhances an individual’s intrinsic motivation and fulfillment, but can also lead to lasting mental health benefits in the long term.

From a reading perspective, positive psychology suggests that positive psychology works can have a positive impact on mental health by stimulating positive emotions, self-identity, and a sense of social support in readers. From a reading perspective, proponents of the theory of positive psychology argue that reading works related to positive psychology can produce multiple benefits psychologically [16]. Several studies have shown that readers are able to feel a positive emotional impact through exposure to positive thoughts and emotional expressions, which can enhance psychological well-being and satisfaction. These works often help individuals to better cope with daily stresses and challenges by encouraging them to utilize their strengths, enhancing their sense of self-identity, and strengthening their social support networks. Further research has also pointed out that reading about positive psychology can also help individuals build greater psychological resilience, i.e., return to normal more quickly in the face of adversity. This positive psychological resilience not only helps individuals to perform well in their personal lives, but also to better maintain social and interpersonal relationships [17]. some literary works have been shown to have positive psychological effects in the context of positive psychology. Through plot, characterization, and emotional expression, these works stimulate readers’ inner positive emotions and psychological resources, thus promoting individuals’ mental health and lifestyle improvement [18]. For example, Yang’s [19] study found that reading positive psychology works can enhance people’s sense of self-identity, emotional regulation and sense of social support, and thus improve their mental health. Therefore, the reading of positive psychology works is not only a recreational activity, but also an effective way of mental health promotion. This is because the reading of literature can be an effective method of promoting students’ mental health and well-being in a school setting. Liu’s [20] study demonstrated that positive literature reading can be used as a therapeutic approach to mental health that can reduce depressive symptoms and improve well-being, which in turn is expected to further enhance students’ quality of life and well-being.

Positive psychology has become an important area of research by investigating ways in which individuals can realize their potential and enhance their well-being and mental health. Whereas, previous research in positive psychology has usually focused on investigating individuals’ emotional states, levels of mental health, and the factors that influence them. Researchers have assessed participants’ emotional states and mental health indicators by using various psychometric tools and instruments, such as psychological questionnaires and mood logs. In addition, some intervention studies have received extensive attention, such as verbal counseling and emotion regulation skills training [21], which are interventions designed to help individuals enhance their positive emotions and mental health. However, despite the remarkable progress in positive psychology in terms of emotion investigation and intervention research, there is a relative lack of in-depth exploration of literary reading in this area. Literature reading, as a process of deep thinking, is believed to have a significant impact on individuals’ mental health and emotional states, but its specific effects and its association with positive psychology remain under-explored [22].

Although there have been studies proving the positive effects of positive psychology works on mental health, with the diversification of social life and the development of information technology, people’s ways of reading literature have become increasingly diverse [23]. In addition to traditional paper-based reading methods, emerging reading methods such as e-reading, audio reading, and video reading are increasingly favored by people. However, different reading styles may have different effects on mental health, but there is a lack of systematic research on the specific effects of different reading styles on college students’ mental health. Therefore, this study aims to investigate the effects of different reading styles based on positive psychology (including paper reading, e-reading, audio reading, and video reading) on the mental health and quality of life of college students, and to further analyze their effects on sleep quality and quality of life, so as to provide more effective interventions and suggestions for college students’ mental health.

Purpose and significance of the study: this study aims to explore the specific effects of different reading styles on the mental health and quality of life of college students, based on positive psychology. With the increase in mental health problems among college students and the growing prevalence of issues such as anxiety and depression, positive psychology has gained attention as an important psychological intervention that emphasizes the improvement of mental health through the promotion of positive emotions and positive thoughts. However, due to the diversity of reading styles, there is a lack of systematic research on these effects. Therefore, this study aimed to explore the specific effects of different reading styles on the mental health and quality of life of college students for the purpose of providing more effective interventions and recommendations for improving their mental health.

## Participants and methods

### Participants

#### Participant recruitment and selection

Participant recruitment began on March 20, 2024 and lasted until April 30th. We chose four universities in the Shanghai area as the study sites, which included (Shanghai University of Political Science and Law, Shanghai University of Physical Education, East China Normal University, and Shanghai University). The target group was students enrolled in these universities, mainly because college students generally face academic stress and mental health challenges.

#### Questionnaire instruments and platform

We used the Self-Assessment Anxiety Scale (SAS) and Self-Assessment Depression Scale (SDS) as the main psychometric instruments. These scales are widely used to assess individuals’ anxiety and depression levels. The questionnaire survey was conducted through an online platform, Questionnaire Star, which facilitated questionnaire distribution, retrieval, and data management.

#### Questionnaire distribution and recovery

A total of 2915 questionnaires were distributed to the participants, which included basic personal information, and the completion of the SAS and SDS scales. The questionnaires were designed with clear language, logical questions and completeness of information in mind to ensure the quality of the data.

#### Data screening and processing

The returned questionnaires were carefully screened and validated to confirm that they contained valid and complete data. Invalid questionnaires mainly consisted of errors in filling out, incomplete information or failure to fill out the scale correctly. These invalid questionnaires were excluded from the analysis to ensure the accuracy and credibility of the follow-up data.

#### Screening for anxiety and depression levels

in the first round of questionnaires, we analyzed participants’ SAS and SDS scores. Participants with high levels of anxiety and depression were identified based on scores above the normal range. These participants were included in the subsequent experimental intervention group for assessment and analysis of intervention effects.

#### Ethical review and informed consent

all participants received a detailed description of the experiment, including its purpose, procedures, risks, and benefits, before participating in the experiment. Each participant signed an informed consent form confirming their voluntary participation and understanding of the experiment. The ethics committee reviewed the study protocol and approved the ethical compliance of the experiment (approval number SH20240126). Through the above steps, we ensured that the data sources of the study and the participant selection process were scientifically sound and transparent. These measures not only helped to ensure the quality of the data, but also protected the rights and interests of the participants and the legitimacy of the study. The results of the survey are presented in **Table 1**.

**Table 1:** First round of scale surveys.

Statistics	Send out	strike out	recall	validity	recovery rate s (%)	SAS&SAS≥50
(n)	2915	55	2860	2860	98%	820

### Experimental design

**Fig 1:**
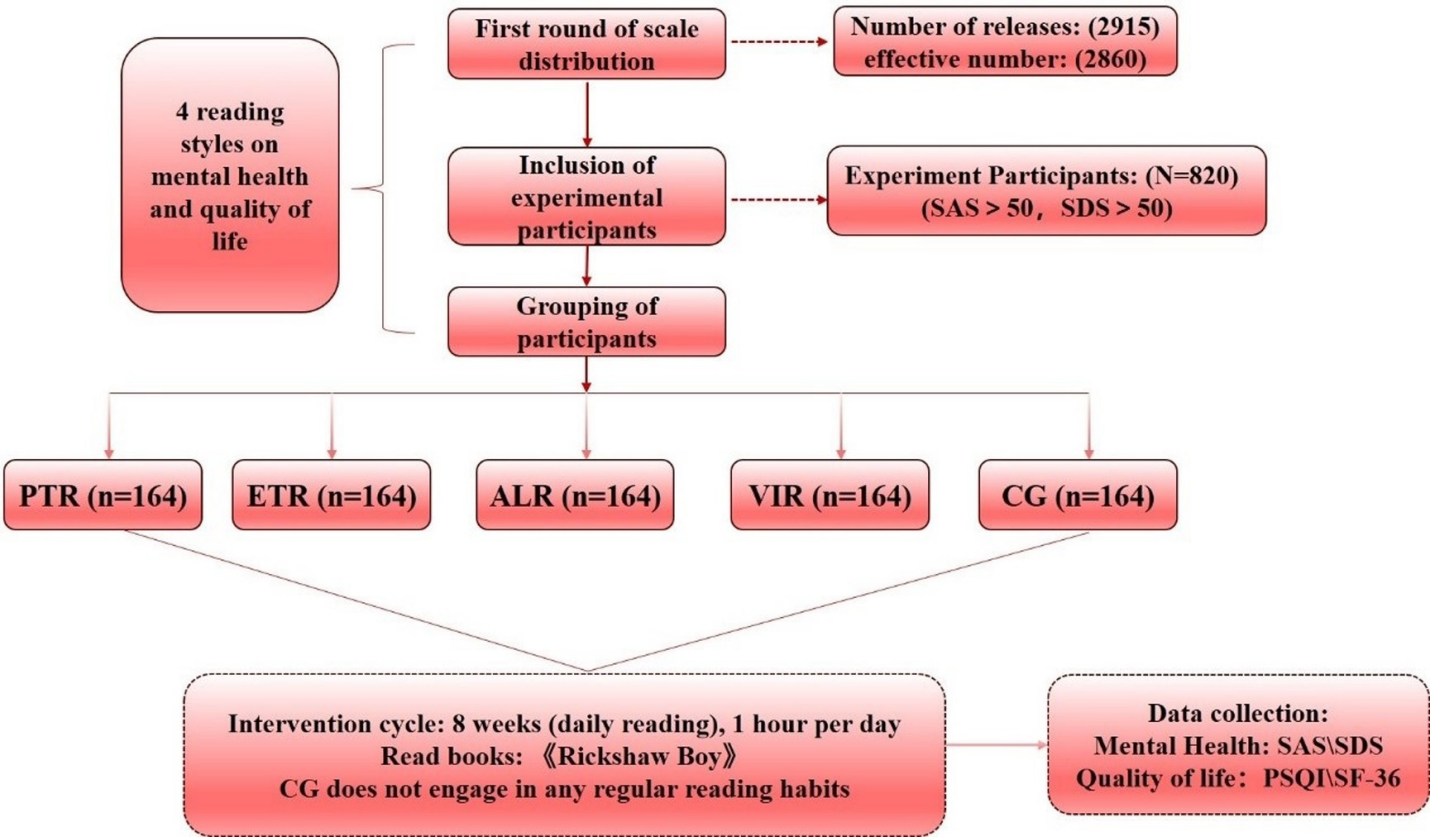
Overall flowchart of the experiment and the content. PTR: paper text reading, ETR: electronic text reading, ALR: audio listening reading, VIR: video image reading, CG: control group.

College students suffering from anxiety and depression levels were randomized in equal proportions into five groups (Paper text reading), (Electronic text reading), (Audio listening reading), (Video image reading), (Control group) (**Table 2**) and an 8-week intervention of literary reading with stories selected from Lao She’s Camel Xiangzi, one of the famous works of modern literature [24]. The stories were selected based on three criteria; (1) positive psychology themes, and (2) participants were given a total of eight chapters (1 per week). Each story contained at least one positive psychology theme, but some stories contained more than one theme. These themes included gratitude, compassion, character strengths, positive thinking, empathy, forgiveness, responsibility, humility, perseverance, and justice. The specific experimental process and protocol are shown in **Fig 1**.

**Table 2:** Statistical grouping of subjects’ information (Mean±SD).

	PTR	ETR	ALR	VIR	CG
n	164	164	164	164	164
Anxiety score	58.21±4.42	59.11±4.37	58.45±3.46	59.29±2.15	58.81±4.17
Depression score	59.67±4.07	59.47±4.01	58.38±3.35	58.29±4.52	58.26±3.92

Note. PTR: paper text reading, ETR: electronic text reading, ALR: audio listening reading, VIR: video image reading, CG: control group

## Methods

### Mental health measurement modalities

#### Self-Assessment Anxiety Scale (SAS)

The Self-Assessment Anxiety Scale (SAS) was developed by William Zung in 1971 (42) and is intended to be widely used among college students for the assessment of anxiety states [25]-. The scale consists of 15 positively rated items and 5 negatively rated items. Each item is rated on a four-point scale: 1 for “none or very little of the time”; 2 for “some of the time”; 3 for “most of the time”; and 4 for “The main statistical measure of the SAS is the total score (standardized score). The total score is calculated by adding the scores of all items, multiplying by 1.25, and rounding to the nearest whole number to obtain the standardized score. Standard scores below 50 are considered normal, while higher standard scores indicate more severe anxiety symptoms. The reliability of the scale was good, with a retest reliability of 0.82. The Cronbacha’s alpha for this study’s scale was 0.976.

#### Self-Rated Depression Scale (SDS)

The Self-Rating Depression Scale (SDS) was originally created by William Zung in 1965 [26]. It consists of 10 positively rated items and 10 negatively rated items. Each item is rated on a four-point scale: 1 for “none or very little of the time”; 2 for “some of the time”; 3 for “most of the time”; 4 for “most or all of the time”; and 5 for “most or all of the time”. “The main statistical indicator of the SDS is the total score. The crude score was multiplied by 1.25 and rounded to the nearest whole number to generate a standardized score, where the crude score was obtained by adding up the scores of all items in the questionnaire. Standard scores below 50 were considered normal, while higher standard scores indicated more severe depressive symptoms. The reliability of the scale was good with a retest reliability of 0.83. The Cronbacha’s alpha for this study scale was 0.82.

### Quality of life measurement modalities

#### Pittsburgh Sleep Quality Index (PSQI)

The Pittsburgh Sleep Quality Index (PSQI) was developed and revised by several scholars [27]. It is designed to assess the quality of sleep of college students during the past month. The scale contains 18 self-assessment items covering seven areas: subjective sleep quality, sleep duration, sleep efficiency, sleep disorders, use of hypnotic medication, and daytime dysfunction. Each item is scored on a scale ranging from 0 to 3, and all item scores are summarized to form a total PSQI score. The higher the total score, the worse the quality of sleep. Some studies have used a PSQI total score greater than or equal to 8 as a criterion for poor sleep quality [28]. The scale has good reliability and validity, with a retest reliability of 0.86. The Cronbacha’s alpha for this study scale was 0.861.

#### Short Form 36 Health Survey Questionnaire (SF-36)

The SF-36 (Short Form 36 Health Survey) is a universal scale for evaluating health-related quality of life, developed by the U.S. Bureau of Medicine Research Group [29]. The scale consists of eight dimensions that assess quality of life in terms of physical health and mental health. These dimensions include Physical Functioning (PF), Role Physical (RP), Bodily Pain (BP), General Health (GH), Vitality (VT), Social Functioning, SF), Emotional Functioning (Role Emotional, RE), and Mental Health (MH). Based on these eight dimensions, two summary scales were constructed in this study:Physical Composite Score (PCS) and Mental Composite Score (MCS).Generally assessing quality of life below 60 indicates a poor standard of living, and the higher the score the better the quality of life. The reliability of this scale was good, with a re-test reliability of 0.87. The Cronbacha’s alpha for this study scale was 0.92.

#### Quality control of the scale

The researchers received special training to ensure the quality of the survey during the first round of the scale’s distribution phase, and during the second round of the pre- and post-intervention distribution phase. The purposes of the surveys were clearly labeled in the guidelines for the use of the questionnaires. The SAS, SDS, PSQI, and SF-36 have a high degree of reliability and validity and are widely used internationally. To ensure that participants were able to truthfully report their psychological state, responses were anonymous and no private information such as name and address was collected. Participants were asked to complete the questionnaire within 2 days after it was sent out to avoid selection bias that would prolong the study period. In addition, to ensure the validity of the reading intervention, the researchers were asked to take ownership of the experimental process and to understand what needed to be done during the intervention, when to start and end the intervention, etc. When analyzing and processing the statistical data after the reading intervention, the researchers strictly followed the principles of truthfulness and objectivity.

#### Statistical analysis

SPSS 26.0 software was used for data processing and analysis. All indicators were analyzed by descriptive statistics and expressed using the mean plus or minus standard deviation. Paired-samples t-test was used for within-group comparisons before and after the intervention, while comparisons between groups were analyzed by ANOVA using the difference [30]. The difference value is also the value of the change after the intervention minus the change before the intervention in different reading styles, so that the statistics better reflect the changes brought about by the effect of the intervention between groups, and the significance level was set at 0.05 in the statistical analysis, with a P-value of less than 0.05 being regarded as significant.

## Results

### Mental health outcomes before and after 4 literary reading interventions based on positive psychology

**Table 3** shows that, the within-group changes in the mental health of college students before and after the intervention of the 4 reading styles. Anxiety results showed that the anxiety scores of reading styles of PTR and ALR were very significantly lower after the intervention than before the intervention (P < 0.01), the anxiety scores of reading styles of VIR were significant after the intervention than before the intervention (P < 0.05), and the anxiety scores of reading styles of ETR and CG were not significantly different before and after the intervention (P > 0.05). Depression results showed that the anxiety scores of reading styles of PTR and ALR were very significantly lower after the intervention than before the intervention (P < 0.01), the anxiety scores of reading styles of ETR were significantly pre-intervention after the intervention (P < 0.05), and the anxiety scores of reading styles of VIR and CG were not significantly different before and after the intervention (P > 0.05).

**Table 3:** Within-group comparison of four reading styles on mental health (Mean±SD).

Variant	Groups	N	Pre-test	Post-test	t	P
Anxiety	PTR	164	58.21±4.42	51.63±3.56	7.106	0.000
	ETR	164	59.11±4.37	57.23±4.10	10.825	0.053
	ALR	164	58.45±3.46	53.24±3.23	36.106	0.010
	VIR	164	59.29±2.15	55.72±3.11	10.702	0.049
	CG	164	58.81±4.17	58.34±5.34	2.956	0.126
Depression	PTR	164	59.67±4.07	52.46±3.13	37.540	0.000
	ETR	164	59.47±4.01	55.72±4.46	9.151	0.031
	ALR	164	58.38±3.35	52.16±4.03	27.142	0.000
	VIR	164	58.29±4.52	56.46±3.21	12.212	0.092
	CG	164	58.26±3.92	58.57±4.74	-2.468	0.420

Note. P< 0.05 indicates a significant difference and P < 0.01 indicates a highly significant difference. PTR: paper text reading, ETR: electronic text reading, ALR: audio listening reading, VIR: video image reading, CG: control group

The **Table 4** shows that the differences between the groups before and after the intervention of the four reading styles on the mental health of college students, and the results show that At the level of anxiety, all four reading styles, PTR, ETR, ALR, and VIR, had significant changes for CG (P < 0.05), and the effect of PTR reading style was more significant, followed by ALR reading style, and there was no difference in the effect of ETR and VIR on anxiety improvement (P > 0.05). At the depression level, all four reading modalities, PTR, ETR, ALR, and VIR, had significant changes for CG (P < 0.05), and the effect of ALR reading modality was more significant, followed by PTR reading modality, and there was no difference in the effect of ETR and VIR on the improvement of depression (P > 0.05).

**Table 4:** Between-group comparison of four reading styles on mental health (Mean±SD).

Variant	Groups	Groups	Difference between mean values	P
Anxiety	PTR	ETR	-4.333	0.000
		ALR	-1.775	0.000
		VIR	-3.720	0.000
		CG	-6.362	0.000
	ETR	PTR	4.333	0.000
		ALR	2.558	0.000
		VIR	0.613	0.075
		CG	-2.028	0.000
	ALR	PTR	1.775	0.000
		ETR	-2.558	0.000
		VIR	-1.944	0.000
		CG	-4.586	0.000
	VIR	PTR	3.720	0.000
		ETR	-0.613	0.075
		ALR	1.944	0.000
		CG	-2.642	0.000
Depression	PTR	ETR	-2.600	0.000
		ALR	.497	0.034
		VIR	-2.767	0.000
		CG	-6.105	0.000
	ETR	PTR	2.600	0.000
		ALR	3.097	0.000
		VIR	-0.167	0.474
		CG	-3.504	0.000
	ALR	PTR	-.497	0.034
		ETR	-3.097	0.000
		VIR	-3.264	0.000
		CG	-6.602	0.000
	VIR	PTR	2.767	0.000
		ETR	0.167	0.474
		ALR	3.264	0.000
		CG	-3.337	0.000

Note. P< 0.05 indicates a significant difference and P < 0.01 indicates a highly significant difference. PTR: paper text reading, ETR: electronic text reading, ALR: audio listening reading, VIR: video image reading, CG: control group

### Results of sleep quality before and after intervention of 4 types of literature reading based on positive psychology

Changes in sleep quality before and after intervention, the **Table 5** shows, within-group changes before and after the intervention of the 4 reading styles on college students’ sleep, the results show that the four reading styles of PTR, ETR, ALR, and VIR had very significantly lower sleep quality scores after the intervention than before the intervention (P < 0.01), and there was no significant difference in the sleep quality scores of the CG’s reading styles before and after the intervention (P > 0.05).

**Table 5:** Within-group comparison of four reading styles on sleep quality (Mean±SD).

Groups	n	Pre-test	Post-test	t	P
PTR	164	11.23±0.54	5.35±0.40	72.188	0.000
ETR	164	12.35±1.10	6.13±0.30	71.039	0.000
ALR	164	12.34±0.35	5.65±0.33	128.936	0.000
VIR	164	11.47±0.48	5.67±0.46	94.677	0.000
CG	164	12.60±1.90	12.20±1.70	3.848	0.361

Note. P< 0.05 indicates a significant difference and P < 0.01 indicates a highly significant difference. PTR: paper text reading, ETR: electronic text reading, ALR: audio listening reading, VIR: video image reading, CG: control group

The **Table 6** shows that the differences between groups before and after the intervention of the four reading styles on the mental health of college students, the results show that PTR, ETR, ALR, VIR four kinds of reading before and after the intervention of sleep quality have significant changes for CG (P < 0.05), and PTR reading mode effect is higher significant, ETR reading mode is significantly the lowest, ALR and VIR for the improvement of sleep quality effect does not have a difference in the change (P > 0.05).

**Table 6:** Between-group comparison of four reading styles on sleep quality (Mean±SD).

Groups	Groups	Difference between mean values	P
PTR	ETR	-.759	0.000
	ALR	-.324	0.084
	VIR	-.574	0.092
	CG	-6.937	0.000
ETR	PTR	.759	0.000
	ALR	.435	0.000
	VIR	0.185	0.048
	CG	-6.178	0.000
ALR	PTR	.324	0.084
	ETR	-.435	0.000
	VIR	-.250	0.076
	CG	-6.613	0.000
VIR	PTR	.574	0.092
	ETR	-0.185	0.048
	ALR	.250	0.076
	CG	-6.363	0.000

Note. P< 0.05 indicates a significant difference and P < 0.01 indicates a highly significant difference. PTR: paper text reading, ETR: electronic text reading, ALR: audio listening reading, VIR: video image reading, CG: control group

### Positive psychology-based quality of life results before and after the intervention of 4 types of literature reading

The **Table 7** shows, the within-group changes in quality of life of college students before and after the intervention of the 4 reading styles, the results show that the quality of life- (PCS) scores after the intervention of the four reading styles of PTR, ETR, ALR, and VIR were very significantly higher than the pre-intervention (P < 0.01), and there was no significant difference in the quality of sleep scores before and after the intervention of the CG’s reading styles (P > 0.05), the Quality of life- (MCS) scores were very significantly higher after the four reading styles intervention for PTR, ETR, ALR, and VIR than before the intervention (P < 0.01), and there was no significant difference in CG’s reading style sleep quality scores before and after the intervention (P > 0.05).

**Table 7:** Within-group comparison of four reading styles on quality of life (Mean±SD).

Variant	Groups	Pre-test	Post-test	t	P
PCS	PTR	71.20±2.31	78.34±3.14	-22.449	0.000
	ETR	70.52±3.16	76.54±1.45	-16.521	0.000
	ALR	71.17±2.65	78.20±2.17	-24.896	0.000
	VIR	70.60±1.82	76.31±1.38	-21.422	0.000
	CG	71.58±2.15	71.88±1.85	0.355	0.725
MCS	PTR	72.54±1.37	78.48±1.66	-38.755	0.000
	ETR	71.72±2.45	79.60±2.51	-38.755	0.000
	ALR	72.30±3.16	79.17±3.15	-37.285	0.000
	VIR	72.67±2.93	78.98±2.68	-21.341	0.000
	CG	71.70±1.87	71.85±2.01	-2.321	0.028

Note. P< 0.05 indicates a significant difference and P < 0.01 indicates a highly significant difference. PCS: Physical composite score, MCS: mental Composite Score, PTR: paper text reading, ETR: electronic text reading, ALR: audio listening reading, VIR: video image reading, CG: control group

The **Table 8** shows, the differences between groups before and after the intervention of the four reading styles on the quality of life of college students, the results show that PTR, ETR, ALR, VIR four reading styles before and after the intervention of quality of life ‐ (PCS) scores have a significant change for CG (P < 0.05), and the effect of PTR and ETR reading styles is more significant. And there was no differential change between. The quality of life- (MCS) scores before and after the intervention of the four reading styles PTR, ETR, ALR, and VIR all had significant changes for CG (P < 0.05), and there was no difference in the effect of the intervention between PTR and ETR, and there was no difference in the effect of the intervention between ALR and VIR.

**Table 8:** Between-group comparison of four reading styles on quality of life (Mean±SD).

Variant	Groups	Groups	Difference between mean values	P
PCS	PTR	ETR	1.697	0.000
		ALR	-0.088	0.763
		VIR	1.975	0.000
		CG	6.753	0.000
	ETR	PTR	-1.697	0.000
		ALR	-1.785	0.000
		VIR	0.278	0.345
		CG	5.056	0.000
	ALR	PTR	0.088	0.763
		ETR	1.785	0.000
		VIR	2.063	0.000
		CG	6.841	0.000
	VIR	PTR	-1.975	0.000
		ETR	-0.278	0.345
		ALR	-2.063	0.000
		CG	4.778	0.000
MCS	PTR	ETR	3.265	1.000
		ALR	-2.147	0.000
		VIR	-2.065	0.000
		CG	5.999	0.000
	ETR	PTR	4.142	1.000
		ALR	-2.147	0.000
		VIR	-2.065	0.000
		CG	5.999	0.000
	ALR	PTR	2.147	0.000
		ETR	2.147	0.000
		VIR	0.082	0.803
		CG	8.147	0.000
	VIR	PTR	2.065	0.000
		ETR	2.065	0.000
		ALR	-0.082	0.803
		CG	8.064	0.000

Note. P< 0.05 indicates a significant difference and P < 0.01 indicates a highly significant difference. PCS: Physical composite score, MCS: mental Composite Score, PTR: paper text reading, ETR: electronic text reading, ALR: audio listening reading, VIR: video image reading, CG: control group

## Discussion

### A positive psychology-based analysis of 4 reading styles affecting college students’ mental health

Our study found that college students’ anxiety and depression levels improved significantly with four different reading modality interventions. Specifically, the PTR and ALR reading modalities showed significant reductions in anxiety and depression scores, and the VIR modality showed significant improvements in anxiety scores. the ETR and CG (traditional reading) modalities showed smaller or non-significant effects on anxiety and depression improvements. When comparing the effects of the different reading modalities together, the PTR reading modality showed the most significant improvement in anxiety, while the ALR reading modality had the most significant effect on depression improvement. This finding is consistent with Zhang’s [31] study, who found that paper books often make people feel the tactile sensation of the texture of the paper, while audio content can create an emotional atmosphere through the conveyance of sound, and these factors work together to motivate people to engage more deeply with the work, which in turn triggers more profound emotional resonance and reflection. Further analysis reveals that the reason why paper text and audio reading methods can be so effective may stem from the fact that they stimulate people’s imagination and emotional expression. Through Macdonald’s [32] study, it was shown that imaginative activities during the reading process could deepen the understanding and experience of the textual content, resulting in a more profound emotional experience. This further emphasizes the importance of choosing a reading style that suits one’s needs. Therefore, through paper text and audio reading methods, people can not only enhance their reading experience, but also improve anxiety symptoms, thus promoting mental health [33]. This is because positive psychology is read in a way that focuses on personal strengths, positive emotions, and experiences aimed at fostering a sense of well-being and psychological resilience [34]. By emphasizing personal strengths and positive emotions, positive psychology helps people to better adapt to challenges, overcome difficulties, and better cope with various stresses and negative emotions in their lives [35].

Our study also found that VIR approach can also significantly improve anxiety levels, albeit slightly less effectively than paper-based text and audio reading. However, video reading also has a higher degree of variability and interactivity, and readers can obtain more information through visual elements, which contribute to the attractiveness and comprehension of the reading [36]. Therefore, our study shows that video reading can reduce anxiety symptoms to a certain extent, especially for those who are not interested enough or not very good at text and audio reading. Therefore, video reading, as an emerging reading method, also has an important role in mental health promotion, especially in the digital reading environment, which provides people with a new reading choice and meets the reading needs of different groups of people. However, this study found that reading by audio reading method did not improve college students’ anxiety. This may be due to the fact that the audio reading method does not provide a similar sense of immersion and imagination as paper-based text reading. It is not a good choice of method for improving anxiety, and in summary, choosing the right reading method for you is crucial for improving anxiety. Paper-based text reading and audio reading are better able to trigger imagination and emotional resonance, thus having a significant improvement effect on college students’ anxiety. Therefore, encouraging college students to choose text reading and audio reading methods will help improve their mental health and reduce anxiety symptoms.

In addition, this paper found that the e-text reading approach was slightly less effective than PTR and ALR in intervening on depressive symptoms, which may be due to the lack of tactile and audio emotional experience in e-text reading compared to traditional paper text reading. This is consistent with the study of Floyd [37], who found that depressed patients often face a state of physical and mental exhaustion, and they may feel tired, helpless, and frustrated, and may even experience physical discomfort, in which their perception of external stimuli and emotional experiences may be reduced, which means that they are more in need of sensory-rich experiences to alleviate their emotional distress. However, electronic text reading often fails to provide the sense of touch and the texture of paper that traditional paper books have, and it also fails to bring about the sound experience that audio reading brings. This prevents depressed patients from enjoying the pleasurable feelings that accompany physical books during reading, thus reducing the likelihood that they will obtain psychological relief through reading. Therefore, the electronic text reading method is not as effective as paper text reading and audio reading. In addition, our study found that the video reading method had no effect on depression improvement, which is different from the previous study by Pan [38], in which a number of previous studies had shown that viewing video content can enhance an individual’s mood state, thereby helping to alleviate mild depressive mood. Our study found that video reading had less of an effect compared to text reading, which may be related to a possible lack of the depth of thought and introspection that text reading has. Depressed patients often need to resolve their inner distress through deep reflection and emotional empathy, while video reading may not provide enough space and time for them to engage in self-reflection and emotional adjustment [39]. Positive psychology emphasizes the use of positive emotions, personal strengths, and positive experiences to enhance individual mental health and well-being Positive psychology-based literature reading has a positive effect on improving the mental health of college students, but when dealing with anxiety and depression, it is necessary to choose the appropriate reading method to avoid the influence of negative emotions, so as to better enhance mental health.

### A positive psychology-based analysis of four reading styles affecting college students’ quality of life

Our study found that college students’ sleep quality significantly improved with four different reading style interventions. Specifically, all four reading styles, PTR, ETR, ALR, and VIR, significantly reduced students’ sleep quality scores after the intervention. Further comparison of the effects between the reading styles revealed that the PTR reading style was the most effective in improving sleep quality. These results further confirm the effectiveness of positive psychology literature reading in improving sleep. Of particular note is the higher significance of the effect of the PTR reading style. This may be due to the fact that the paper text reading approach is closer to the traditional reading experience and can provide a more comfortable and immersive reading environment, which helps to relax the brain and body and mind, thus promoting the improvement of sleep quality [40]. This is in line with a previous related study by Zhang [41]. His study found a correlation between literature reading and sleep quality. By immersing themselves in literature, readers can get rid of the stress and anxiety of real life and enter a relaxed and quiet mental state, which helps to fall asleep and improve the depth of sleep. Promoting the improvement of college students’ sleep quality will bring multiple benefits. In addition, good sleep quality can improve the attention and concentration of college students, which can help improve learning efficiency and academic performance [42]. Therefore, this study further confirms the important role of positive psychology literature reading on college students’ sleep quality and provides an easy and effective intervention to promote college students’ physical and mental health.

In addition, our results found that the quality of life of college students significantly improved under four different reading style interventions. Specifically, the four reading styles, PTR, ETR, ALR, and VIR, significantly improved students’ physical health and mental health composite scores (PCS) after the intervention. Conventional reading style (CG) had no significant effect on quality of life improvement. Further comparison of the effects between the reading styles revealed that the PTR and ETR reading styles were more effective in improving the physical health scores, while there was no significant difference in the effects of ALR and VIR on the mental health scores. Therefore, In our study, we found that the quality of life-PCS (physical health subscale) and quality of life-MCS (mental health subscale) scores of college students were significantly improved through the literature reading intervention of positive psychology. This implies that literature reading not only helps to improve the physical health of college students, but also enhances their mental health, which comprehensively improves the overall quality of life. This is consistent with Egert’s [43] study that by immersing themselves in literature with positive psychology, readers can experience the lives of different characters and feel emotional resonance and empathy, thus promoting their own emotional regulation and psychological growth. This emotional experience and thinking process can improve an individual’s mental health, reduce negative emotions such as anxiety and depression, and enhance psychological resilience and coping ability, thereby improving the quality of life [44]. This study further confirms the positive impact of positive psychology literature reading on quality of life. This finding not only provides an easy and effective way for college students to enhance their quality of life, but also provides new ideas and approaches for mental health interventions. By promoting and applying this positive psychology literature reading approach, it can further promote the physical and mental health of college students and improve their overall quality of life. And it provides a reasonable choice for college students to choose the appropriate way of reading.

Therefore, this paper examines different reading styles through positive psychology and finds that it can significantly improve the mood and quality of life of college students. Paper text reading and audio reading helped to reduce anxiety levels and enhance mental health and well-being. Visual reading, although slightly less effective, provides interactivity and variability that is suitable for college students in a digital reading environment. Choosing the right type of reading is critical to improving anxiety, and these studies provide effective mental health strategies and reading options for college students.

#### Strengths

This is a first-of-its-kind study that examines the effects of different reading styles on the mental health and quality of life of college students, based on a positive psychology perspective, and fills a research gap in a related field. The study involved four different reading styles, including paper text reading, electronic text reading, audio listening and video image reading, and explored the impact of reading styles on mental health and quality of life from multiple perspectives. The study adopted positive psychology as an important psychological intervention, emphasizing the improvement of mental health through the promotion of positive emotions and positive thoughts, and providing more effective interventions and suggestions for the mental health of college students.

#### Limitations

sample limitations, lack of detail in the experimental design, and possible subjectivity in the interpretation of results. Future research could further improve sample selection, expand the scope of the study population, and increase the diversity of the sample in order to increase the generalization of the findings. In addition, future studies could enhance the rigor of the experimental design by describing the experimental steps and control variables in more detail to ensure the reliability of the findings.

## Conclusion

This study examined how various reading styles rooted in positive psychology affect college students’ mental health and quality of life. Paper-based text and audio reading significantly reduced anxiety levels, likely due to their tactile and emotional engagement benefits, fostering deeper emotional resonance and reflection. Paper text reading notably enhanced sleep quality by creating a relaxing, immersive environment. In contrast, electronic text reading proved less effective than paper and audio formats, possibly due to its lack of tactile and immersive qualities. Video reading did not improve depression symptoms compared to text reading, possibly due to its limited capacity for introspection. These findings underscore the benefits of paper and audio reading for mental health enhancement, offering guidance for college students seeking effective reading strategies aligned with positive psychology principles. These findings have important academic and practical implications for promoting mental health and improving quality of life among college students.
